# Structured expert elicitation for long-term survival outcomes in health technology assessment: a systematic review

**DOI:** 10.1186/s12911-025-03221-2

**Published:** 2025-10-21

**Authors:** Jessica E. Forsyth, Lesley Uttley, Ruth Wong, Shijie Ren

**Affiliations:** Sheffield Centre for Health and Related Research (SCHARR), University of Sheffield, Regent Court, 30 Regent Street, Sheffield, S1 4DA UK

**Keywords:** Structured expert elicitation, Healthcare decision-making, Systematic literature review, Health technology assessment, Survival extrapolation

## Abstract

**Background:**

Extrapolation of immature survival data is integral to health technology assessment (HTA) but is often associated with large uncertainty. Incorporation of expert judgements can help to address this uncertainty, but often these judgements are perceived as being “best guesses” due to a lack of methodological transparency. This review assesses the current implementation and reporting of structured expert elicitation for long-term survival outcomes in the broader literature and recent submissions to the National Institute for Health and Care Excellence (NICE).

**Methods:**

Three literature databases were searched: MEDLINE via Ovid, Embase, and Web of Science. The search algorithm included terms to identify articles which obtained expert judgements for survival extrapolation. A pearl-growing approach was also employed using three seed papers to supplement the electronic searches. To identify recent NICE technology appraisals in oncology, the NICE guidance database was searched.

**Results:**

The search of the broader literature identified six studies which utilised structured expert elicitation for long-term survival outcomes. Four NICE technology appraisals were identified to have used structured expert elicitation between October 2023 and October 2024. The reporting and conduct of elicitation for survival quantities was variable in detail and rigour in both the broader literature and NICE submissions. Despite the requirement for significant resource investment within the process, elicited values are variably used within analyses and are predominantly used as qualitative external validation. At all points throughout the elicitation, including planning, conduct and reporting, there appears to be a considerable lack of technical detail, which in the context of NICE appraisals may hinder full consideration of the elicited values by reviewing committees.

**Conclusions:**

Currently, the methods and limited reporting structures being used to elicit long-term survival outcomes are not fit for purpose. This review highlights key areas for improvement and identifies examples of good practice when conducting structured expert elicitations for long-term survival outcomes.

**Supplementary Information:**

The online version contains supplementary material available at 10.1186/s12911-025-03221-2.

## Background

In health technology assessments, cost-effectiveness analyses often extend over a period significantly greater than the follow-up period of the pivotal trial informing the clinical effectiveness. Survival data is a key input into the cost-effectiveness analysis and due to the limited trial follow-up, requires extrapolation beyond the observed period in order to align with the time horizon of the economic model. Extrapolation of survival data can be performed using standard parametric models, more flexible spline-based methods, or alternatively can incorporate approaches which encompass external data such as registry data [1–4]. However, different statistical models can provide significantly different predictions of survival at later follow-up times, especially in oncology or rare disease settings [5, 6]. To help mitigate the impact of this uncertainty, clinical experts, with expertise on relevant subject matter, are often consulted to help choose the most clinically plausible model for use within the cost-effectiveness analysis [1, 7–9].

Clinical experts are generally asked to provide their judgements on long-term survival via one of two ways: (1) direct selection of an extrapolated model that they believe to be the clinically most plausible, or (2) prediction of survival at a particular time point, which is subsequently used to compare candidate model extrapolations [10, 11]. Both approaches are potentially subject to bias, and any external validation based upon expert judgements is often associated with some scepticism due to the lack of transparency and robustness of the consultation process [12]. 

Structured expert elicitation is an alternative approach to obtain expert judgements. This structured approach explicitly aims to minimise potential biases that are often associated with more informal approaches to expert consultation [13, 14]. Structured approaches represent expert judgements and expert uncertainty as probability distributions and facilitate the representation of a group of experts’ judgements as a single distribution via mathematical or behavioural aggregation. This differs significantly from the aforementioned approaches which often use informal discussions to obtain expert judgement without systematic quantification of uncertainty.

Several structured expert elicitation protocols have been developed such as the: Sheffield Elicitation Framework (SHELF) [15]; modified Delphi method [16]; Cooke’s classical method [17]; Investigate, Discuss, Estimate, Aggregate (IDEA) protocol [18] and the Medical Research Council (MRC) reference protocol [13]. These are provided as illustrative examples rather than an exhaustive or prescriptive list, but each of these protocols generally describe how to conduct an expert elicitation and are described further by Bojke et al. [13]. The protocols outline key steps for the preparation and design of the elicitation such as: expert selection, preparation of an evidence dossier, format/setting of the elicitation, definition of quantities of interest (QoI), statistical training provision to experts, collection of individual judgements, expert group discussion and methods to aggregate multiple experts’ opinions. There are some similarities between the frameworks such as the general consensus on the value of collecting individual expert judgements prior to group discussion in order to minimise potential biases due to anchoring or dominance effects. However, there are also differences such as the inclusion of facilitated group discussion, advocated in the IDEA, SHELF and MRC protocols, in order to allow experts to share reasoning, challenge assumptions and refine estimates [13, 15, 18]. This contrasts with the classical Cooke’s method where this discussion is not an essential component of the elicitation. A comprehensive review of these protocols is also provided by Soares et al., which summarises the similarities and differences between the five approaches [14]. 

These five protocols describe structured elicitation approaches for non-specific quantities, though the MRC reference protocol is largely considered in the context of health-care decision-making [13]. Time-to-event data, used when performing survival extrapolation, differs from other types of data, namely due to the presence of censoring and an inherent one-to-one relationship with the hazard function, this makes its use within elicitations more complex compared with other types of data [19]. Therefore, adaptations to existing elicitation protocols are required to ensure unbiased expert judgements are obtained whilst fully considering the survival data and hazard function. Recently published guidance, in the form of a NICE technical support document (TSD) (TSD 26) describing expert elicitation for long-term survival outcomes, discusses the first bespoke methodology for eliciting survival quantities [20].

While specific methods guidance relating to the elicitation of long-term survival outcomes has not been available until now, there are several instances of structured methods being used for long-term survival outcomes with significant adaptation and variability in design [7, 8, 21]. This review provides a comprehensive description of existing use cases of structured expert elicitation for long-term survival outcomes in the broader literature as well as recent use cases within oncology NICE health technology assessments. By reviewing the current implementation of structured expert elicitation for long-term survival outcomes, and the reporting quality of the structured expert elicitation exercises, this review helps to identify key areas for improvement. This review also highlights good examples of implementation and elicitation design which could help influence future elicitations and improve their credibility and transparency, thus enhancing the utility of these approaches within health technology appraisals.

## Methods

A protocol for the search of the wider literature is available on the Open Science Framework [22], but all methodological components relating to the wider literature search and the review of NICE technology submissions are outlined within this manuscript.

### Information sources & search strategy

For the search of the wider literature, three databases were searched: MEDLINE via Ovid, Embase and Web of Science. These databases were chosen to ensure broad coverage of the literature whilst ensuring that a focus on healthcare decision-making was retained. All databases were searched from inception until July/August 2024.

A search algorithm was designed for the database search with an Information Specialist (RW) using broad keywords for “expert elicitation” and synonyms combined with keywords for “survival” (see supplementary). The search was adapted across the three databases including a limit to English only publications.

To ensure that all relevant literature was identified, we used a pearl-growing approach (also known as “snowballing”), whereby citations of a primary seed paper are screened for relevance and where new and relevant articles are found, the process is repeated within those papers. We used three seed papers including: Ayers et al. [7], Cope et al. [8] and Willigers et al. [23] via the web version of citationchaser on the 21st of August 2024 [24]. The three seed papers were identified via previous hand-searching of the literature. All included studies were subject to hand-searching of the reference list for any missed relevant publications.

Peer-reviewed publications may not fully capture how structured expert elicitation is applied in the real-world decision-making context. To address this gap, we conducted an additional targeted review of recent NICE oncology submissions published within the period 1st October 2023 to 14th October 2024. All documents tagged as relating to “oncology” were eligible for review. These were obtained through the public NICE website using the date and “Technology appraisal guidance documents” filters.

### Study selection

As this is a methodological review, the Sample, Phenomenon of Interest, Evaluation, Research Type (SPIDER) framework was used to define the inclusion and exclusion criteria for the search of the broader literature, Table 1 [25]. 

**Table 1:** Inclusion and exclusion criteria for study review according to the SPIDER framework

Inclusion Criteria	
Sample	Clinical/methodological experts
Phenomenon of Interest	Use of structured expert elicitation in relation to long-term survival outcomes.
Design	Primary empirical studies
Evaluation	Conduct, design, outputs and experience of structured expert elicitations in the context of long-term survival outcomes.
Research Type	Empirical studies published in the English language in peer-reviewed journals.
**Exclusion Criteria**	
Conference abstracts, reviews or discussion pieces	

Studies identified by the database search were exported into an EndNote library and were first screened according to the title and abstract by JEF and SR and any disagreements regarding inclusion status were resolved through discussion with LU. Remaining articles were then subject to full-text review by JEF, with random selection and review by SR.

Any NICE appraisals that had been terminated were excluded from the initial sifting. Only full company submissions were considered for this review, and therefore only appraisals where “Document B” was publicly available were eligible for analysis. The downloaded company submissions sometimes contained redacted information, and this was noted in any cases where this prevented full review of the appraisal.

The appraisals were then screened for relevance (JEF) using two approaches. First, by undertaking keyword searching for the occurrence of elicit*, expert, and extrap* and second, referring to sections within the documentation where structured expert elicitation for survival outcomes is likely to be found and reported. Potentially relevant appraisals identified through either approach were subjected to content review followed by data extraction. Where the reporting and use of structured expert elicitation was unclear, a second reviewer (SR) was consulted to discuss eligibility of the appraisal.

### Outcomes & data extraction

The nature of this review is largely qualitative due to the variability of reporting; therefore, a broad range of outcomes were considered for data extraction. Data items included: first author surname, publication year, clinical area, number of experts, the expert selection process, (base) elicitation framework, elicitation setting (in-person or online), roles listed (e.g., facilitator), declaration of conflicts of interest, details of expert backgrounds/specialties, details of training provided, details of evidence dossier preparation/content, quantities of interest (QoI), individual judgement method (e.g., tertiles, quartiles, roulette), aggregation of expert judgements (e.g., rational impartial observer (RIO) or mathematical aggregation), form of QoI (e.g., range, fitted distribution), rationale for expert judgements provided, discussion of the hazard and discussion of limitations/benefit of expert judgements.

Transparent reporting is a key aspect of conducting structured expert elicitation, and our intention was also to evaluate how clearly the exercises could be understood and replicated from the available documentation. Study authors were therefore not contacted in the event of missing information as this would have introduced information not accessible to typical end-users of these reports, and thus would not reflect the transparency of reporting in the original publication. All data items extracted were recorded within an excel spreadsheet by JEF, and SR checked a random sample of to ensure consistency. Any discrepancies between the data extracted by JF and SR were discussed with LU, and the data item was subsequently re-extracted for the remaining studies.

Relevant NICE technology appraisals were also reviewed for potential inclusion, and narrative summaries of expert involvement, relating to long-term survival outcomes, were generated. The summaries were then used to identify appraisals where structured approaches were implemented and formed the basis of the qualitative assessment of conduct.

### Reporting quality assessment

Clear and comprehensive reporting of elicitation exercises is important to ensure full transparency of the exercise. Reporting was therefore assessed according to the level of description of integral components of elicitation exercises. These components were identified from the aforementioned existing protocols and included the reporting of: the number of experts, conflicts of interests, expert identification process, expert backgrounds, the base elicitation protocol, setting of the elicitation, evidence dossier preparation and provision, QoI definitions, provision of training, details of the elicitation exercise format, method of expert judgement aggregation, expert qualitative rationale and the eventual use of the elicited distributions. The reporting quality for each item was graded as one of the following classes: (1) complete reporting, where the item is described in detail with no outstanding questions; (2) partial reporting, where the item is mentioned but additional clarification is required for complete understanding of the implementation/process; or (3) no reporting, where the item was not discussed within the study/appraisal. The assessment of the reporting quality was made by JEF, where each component was graded as being either completely, partially or not reported. These allocations were discussed with SR and any discrepancies discussed with LU. However, we stress that reporting quality does not necessarily equate to methodological quality of the elicitation exercise.

### Statistical analysis

Due to the qualitative nature of this review, statistical analysis was not appropriate. Where meaningful, quantitative summaries of numerical outcomes are provided using the relevant summary statistics.

## Results

**Fig. 1:**
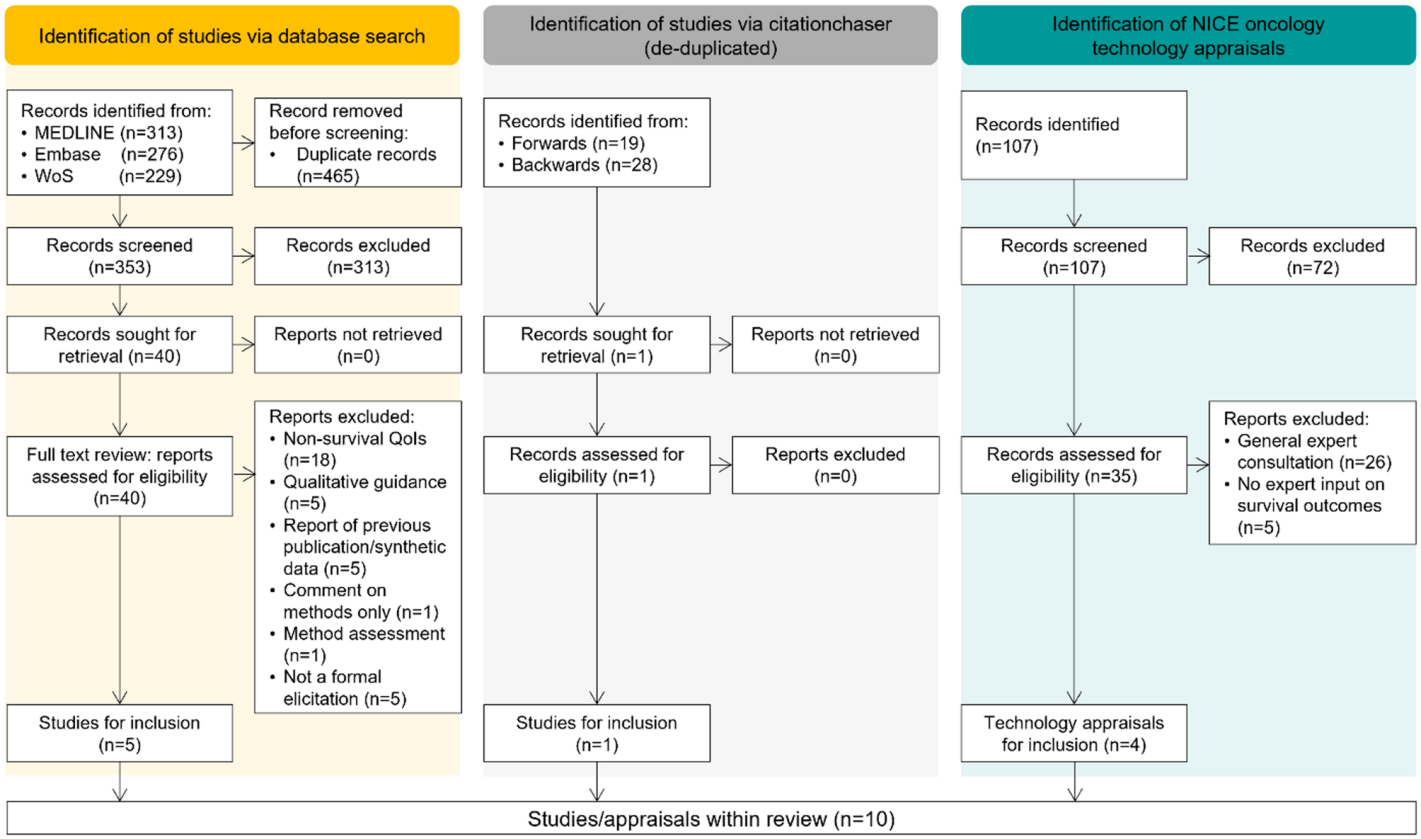
Preferred reporting items for systematic reviews and meta-analyses (PRISMA) diagram of broader literature search and targeted review of recent NICE oncology appraisals. QoI, quantity of interest

Bibliographic database searches retrieved 353 unique articles, of which 313 articles were excluded based on the assessment of non-relevance during title and abstract screening. Searching of the NICE published guidance database identified 107 technology appraisals, of which 72 were not related to oncology or were terminated appraisals. Of the 40 studies and 35 appraisals subject to a full-text review, including those identified from the forward or backward pearl-growing, 6 studies and 4 recent technology appraisals had used structured expert elicitation for long-term survival outcomes, (Fig. 1, see supplementary material for list of included studies and appraisals).

Of the studies identified within the broader literature, all studies were published between 2019 and 2023. Five out of six of the studies were within oncology [7, 8, 21, 26, 27], with the sixth study based in renal disorders [23]. The four NICE technology appraisals (TA), henceforth referred to as ‘appraisals’: TA917 [28], TA954 [29], TA967 [30], and TA975 [31], related to multiple myeloma, B-cell lymphoma, classical Hodgkin lymphoma, and lymphoblastic leukaemia respectively. The four appraisals were all submitted by different companies and received positive guidance for the proposed interventions. Whereas in the six studies identified within the broader literature, two of the studies, authored by Ayers et al. and Cope et al., had significant overlap in contributing authors [7, 8]. 

### Reporting quality of studies/appraisals

**Fig. 2:**
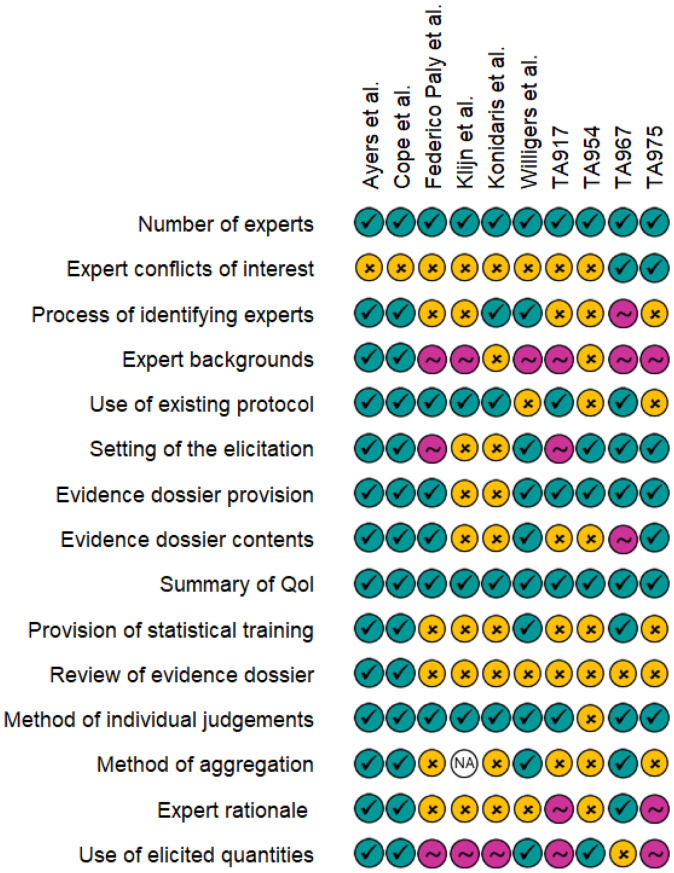
Reporting quality assessment of included studies and appraisals. Teal ticks indicate complete reporting, pink tilde indicates that the item was mentioned but additional details are required for full understanding of the process, and yellow crosses indicate that the item was not reported. TA, technology appraisal; QoI, quantity of interest

For each of the included studies and appraisals, reporting detail of common elements of elicitation exercises was assessed. These were graded as: completely reported; partially reported; or not reported (Fig. 2). It is evident that some items, such as the number of experts or the definition or the quantity of interest are generally well reported, but other items such as expert declarations of conflicts of interest or the process of identifying experts are more variably reported. Expert backgrounds tended to be introduced within the studies, but then not expanded on. The eventual use of the elicited quantities was not always discussed fully within the studies.

### Selection and identification of experts

Studies identified from the broader literature had between one [26] and nine [27] experts, with a median of six experts. These experts were involved in all stages of the elicitation, whereas in the NICE appraisals there were between three [31] and ten [28] clinical experts with variable contributions within the elicitations. For instance in TA975, despite the recruitment of six clinical experts, only five were able to provide their individual judgements due to technical issues, but all experts contributed within the discussions [31]. 

Expert declarations of conflicts of interest were not included within any of the studies identified from the broader literature, and this was similarly not discussed extensively within the appraisals. Only TA967 and TA975 stated that this information was collected but it was not presented within the publicly available documents [30, 31]. However, this information may have been available for the NICE committee and external assessment group during review of the submission via the supporting appendices. Alternatively, conflicts of interest could have been included as part of the expert selection criteria, with only those without conflicts deemed eligible; however, it is unclear whether this was the case. Throughout this review, it is noted that the assessment of the appraisals is limited to the publicly available information and documents, however references to supporting documents (which are not publicly available) is highlighted.

The process of selecting experts was variably reported. Ayers et al. and Cope et al. described the process of selecting experts, largely with a focus on “criteria” for the expertise of participating experts as well as expert experience with the intervention(s) [7, 8]. The study by Konidaris et al., associated with NICE TA592, did not include explicit description of the expert selection process but within Document B of the associated appraisal, it was highlighted that all experts were required to have treatment experience with the intervention and were recruited from the pool of trial investigators [27, 32]. Federico Paly et al., Klijn et al. and Willigers et al. did not include significant information regarding the selection process of the experts [21, 23, 26]. No details of the expert selection process, or attempts to ensure panel diversity were discussed in any of the four NICE appraisals.

The discussion of expert backgrounds or expertise was largely correlated with the level of detail on the expert selection process. Both Ayers et al. and Cope et al. provided detailed, quantitative descriptions of experts, including: proportions of time spent providing direct patient care, numbers of years practicing within disease area and numbers of patients treated within a typical monthly period [7, 8]. For the remaining studies and appraisals, experts were largely described as per their clinical discipline, without any quantification of length of time within the area or length of time practicing.

### Use of existing protocols or frameworks

All identified studies within the broader literature, except that by Willigers et al. [23], used SHELF [15] as the base framework for the design of the elicitations whereas two out of four [28, 29] of the NICE appraisals used the MRC protocol [13], with the two remaining appraisals not referencing specific frameworks. All cases where an existing elicitation protocol or framework was cited, acknowledged the necessity for significant modifications to the original framework, for instance modifications to enable the elicitation of survival at multiple timepoints [8] and the adaptation into what appeared to be a remote format where experts could work through the questions in their own time [21].

### Setting of the elicitation

None of the studies identified from the broader literature appeared to conduct the elicitation in a face-to-face setting, though Klijn et al. and Konidaris et al. did not explicitly report the format of the elicitation [26, 27]. Both Federico Paly et al. and Willigers et al., appeared to have conducted the elicitations remotely, using a questionnaire format, as opposed to a structured discussion [21, 23]. Ayers et al. and Cope et al., again used similar approaches as each other and conducted the elicitations virtually [7, 8]. Three of the four NICE appraisals [28–30] stated that the elicitation took place as part of advisory board meetings and TA954 reported that this was conducted face-to-face. In TA975, the meeting was held virtually but it is unclear whether the meeting was specifically for the elicitation relating to survival outcomes, or whether other issues relating to the submission were also discussed [31]. 

### Provision of an evidence dossier

A key element of structured expert elicitation exercises, as described in multiple existing elicitation protocols, is the construction and provision of an evidence dossier. The evidence dossier helps to summarise all relevant data for participating experts and thus serves to minimise availability bias [13, 15, 18]. Four out of six of the studies in the broader literature recorded that an evidence dossier was provided to the experts prior to the elicitation exercise, and one of these studies, by Federico Paly et al., included the dossier within the supplementary material of the publication [21]. The dossiers typically included summaries of relevant trials and a summary of the purpose of the elicitation. In the evidence dossier compiled by Willigers et al., it was stated that potential extrapolations of the survival data were included, the authors however acknowledged this could have biased experts when making their initial judgements [23]. 

All the NICE appraisals appeared to provide “pre-read” materials to experts ahead of the elicitations however the content of the dossiers appeared to vary considerably. In TA917 and TA967, details of the dossier contents were not provided, but in TA917 it was stated that experts did review the dossier in order to ensure no relevant material was omitted [28, 30]. Both TA954 and TA975 included general descriptions of the dossier contents, but in TA954 it was highlighted that “modelling approaches” were also included. It was not clear whether this related to the extrapolation of survival data or other modelling assumptions [29, 31]. 

The majority of the studies in the broader literature and the NICE appraisals did not describe how the evidence dossier content was sourced. Willigers et al. noted that a systematic literature review was not conducted when sourcing information for the dossier, which posed a potential limitation, by contrast, in TA967 a systematic literature review was used to populate the dossier [23, 30]. 

### Definition of quantities of interest

During the preparation of the elicitation, the quantity of interest (QoI) must be defined clearly such that experts are making judgements of the same quantity.

In all studies identified from the broader literature, survival quantities at multiple landmark timepoints were elicited, and in some cases these quantities were elicited alongside the mean lifetime survival. This was mirrored in all but one [30], of the NICE appraisals. Typically, the chosen timepoints reflected time periods in keeping with the standard progression of the condition of interest, though these time points did largely tend towards more rounded landmarks such as 10 or 20 years with no justification of their choice. Survival appeared to be largely described as a proportion, but in TA975, experts were asked to express the survival as the number of patients who would still be alive out of an initial number of patients at a given time point [31]. 

Ayers et al., in addition to the survival proportion at three time points, asked experts to provide the time at which they believed the survival to be 0%. This value served as an anchor to the experts and helped the experts “consider the potential shape of long-term survival” [7]. 

Most studies did not aim to elicit conditional survival outcomes. However, Ayers et al. and Willigers et al. defined the quantities of interest such that this explicit relationship between multiple time points was considered whilst experts made their judgements [7, 23]. Ayers et al. asked experts, to consider the survival at subsequent time points conditional on their judgements at earlier time points. Whereas Willigers et al. first asked for the non-conditional expert judgement of survival at 10 years and then for the survival proportion at 20 years, based on two pre-chosen conditions: survival at 10 years was 40% and survival at 10 years was 70%. Explicit consideration of conditional survival did not appear to be accounted for in the NICE appraisals.

### Provision of statistical training

The elicitation conducted by Willigers et al. reported statistical training of experts, prior to the making of their judgements [23]. The training encompassed details of how to complete the survey, a summary of common biases and heuristics [33] and descriptions of percentiles and their interpretation. Ayers et al. and Cope et al. appeared to also provide statistical training, though the training by Cope et al. appeared to be in a self-directed format [7, 8]. Out of the four NICE appraisals, only TA967 stated any provision of training [30]. TA967 used the Structured Expert Elicitation Resources (STEER) materials [34], which closely align with the MRC protocol [13], and provided a training question to help familiarise experts with the interface which facilitates the input of their judgements.

The experts in the study by Willigers et al. appeared to make their judgements remotely, however the training was conducted via a one-hour virtual session with experts [23]. In TA967, training materials were provided to experts and they were encouraged to contact the elicitation team if they had queries, it was not stated whether any experts did reach out regarding the content in the training [30]. All other studies and appraisals did not mention the provision of statistical training to experts.

### Elicitation methodology and conduct

Following the provision of training or review of the evidence dossier, experts are required to make their individual judgements for each quantity of interest. In the broader literature, both Ayers et al. and Cope et al. asked experts to provide the upper and lower plausible limits, alongside a most likely value [7, 8]. Federico Paly et al., Klijn et al. and Konidaris et al. all asked for upper and lower limits with a central or mean value [21, 26, 27]. Willigers et al. instead asked experts to provide judgements of the 10th and 90th percentiles, along with the median [23]. All of the studies identified in the broader literature therefore appeared to use a variable interval method, as opposed to a fixed interval method where they would instead make judgements on the probability of the true value falling within pre-defined ranges.

In TA954, it was not clear what method was used when experts were making their individual judgements and how expert uncertainty was captured quantitatively [29]. In TA967 the “chip-and-bin” method was utilised, which was implemented via the STEER materials, however it was not clear whether the bins were predefined or defined by the experts themselves [30, 34]. In TA917 and TA975, experts, as in the broader literature studies, were asked for upper and lower estimates of the quantities of interest, along with a most likely value [28, 31]. 

All but one of the cases discussed within this review had more than one participating expert, therefore aggregation of individual judgements would have been required in the remaining studies or appraisals. How this aggregation was performed was not reported in two of the studies from the broader literature [21, 27]. Of the appraisals, only TA967, which was based on the MRC protocol, described the mathematical linear pooling of the individual expert judgements via the SHELF fitdist function [30]. This was conducted during the advisory board meeting and was discussed by experts during the consensus session.

Both Ayers et al. and Cope et al. explicitly recorded expert rationale during the elicitation [7, 8]. Ayers et al. asked experts to comment on what sources of information they primarily used when making their individual judgements and then also recorded any points in discussion during the consensus meeting where experts disagreed. Experts’ rationale and reasoning were also recorded by Cope et al., again at the stage of the consensus meeting. It did not appear that any of the other studies identified within the broader literature recorded qualitative rationale, or this was not presented within the summary of the elicitation.

In TA917, discussion amongst experts was evident, and it appeared that this took place after the meeting where experts initially provided their judgements [28]. In TA967, as part of the STEER materials, experts were required to provide their judgement rationales via a free text box, these were then collected and discussed at the subsequent advisory board meeting [30]. Within TA967 it was highlighted that the qualitative rationale from experts was useful in providing meaningful insights to the inter-expert variability. In both TA917 and TA967, it appears that the expert rationales were summarised in more detail and available within the submission appendices, but these were not publicly available and so could not be assessed as part of this review. In TA975, again it appeared that opinions and discussions of experts were recorded and available within the appendices, however it was not clear whether this specifically related to the elicitation of the survival outcomes or other issues discussed within the meeting [31]. 

Despite the inherent relationship between the survival function and the hazard function, none of the studies in the broader literature or the identified NICE appraisals appeared to discuss the hazard trend directly, either as rationale for experts, or as a source of validation as recommended in NICE TSD 26.

### Use of elicited quantities

Despite the primary outcome of a structured expert elicitation being to provide a probability distribution summarising expert uncertainty, only three out of the six studies in the broader literature appeared to utilise the resulting expert distributions [7, 8, 23]. In the studies by Ayers et al. and Cope et al. the aggregated probability distribution corresponded to that of the “rational impartial observer” as recommended by SHELF [7, 8, 15]. The aggregated distributions were subsequently used to constrain the fitting of the survival models. Willigers et al. similarly used the elicited distributions within a Bayesian survival analysis setting [23]. The three remaining studies appeared to use the expert judgements for external validation, and helped refine the model choice by excluding any model with extrapolated survival outside of the identified plausible range [21, 26, 27]. This was further supported by the lack of reporting of aggregation methodology for these three studies.

In the NICE appraisals, there were variable uses of the expert-derived distributions or ranges. In TA917, experts provided their individual judgements of the quantities of interest and were also asked to rank different extrapolations of survival in terms of their plausibility, whilst excluding any that did not appear plausible as per the experts’ original judgements [28]. In TA954, the most-likely values, provided by the experts were used to assess validity of the extrapolated curves, but the expert uncertainty did not appear to have been considered during selection of the models [29]. In TA967, experts provided individual judgements which were subsequently mathematically aggregated [30]. Within the appraisal a single, “experts’ preferred distribution” is then referenced, however it is unclear how the distribution from the elicitation contributed to the choice of the preferred model. Finally, in TA975 experts were asked to use their own individual judgements to determine specific extrapolations which were not clinically plausible (due to them falling outside of the experts’ upper and lower range) and their overall preferred extrapolation with regards to clinical plausibility [31]. 

## Discussion

We conducted a systematic literature review to provide a comprehensive overview of the use and reporting of structured expert elicitation for long-term survival outcomes across the broader literature. We also performed an additional targeted review of recent NICE oncology submissions to explore the practical application of structured expert elicitation in real-world decision-making contexts. We found that expert consultation on long-term survival outcomes is common within health technology assessment; however, use of more formal, structured elicitation methodologies appears less frequently documented in the broader literature and NICE technology appraisals. We also found that there is equal variability and lack of reporting detail of elicitation exercises in both the broader literature and recent submissions to NICE.

It was observed that in the broader literature, studies typically used the SHELF framework [15] as a basis for the elicitation design, but often there were considerable modifications to the framework due to the nature of the quantities of interest and the preferred formatting of the elicitation exercises. In two of the NICE appraisals, it appeared that the MRC protocol [13] was instead used as the basis framework, contrasting what was observed in the broader literature.

Within the recently published NICE TSD relating to structured expert elicitation for survival outcomes (TSD 26), the authors discuss the elicitation format and highlight that interaction with experts, both during training and discussion of expert judgements, by a trained facilitator is crucial [20]. This discussion is described to be essential in order to ensure meaningful unbiased estimates with sufficient expert rationale. However, within this review we found several studies that appear to have adapted their elicitation design such that training was self-directed and experts made their judgements remotely without support from the elicitation team. From this review, the choice of elicitation format appears to be a key area for unification and development.

Despite the multiple areas for improvement, both in conduct and reporting of structured expert elicitation, there were several studies which presented clear and comprehensive descriptions of the elicitations. For instance, Ayers et al. and Cope et al. both included detailed quantitative summaries of expert backgrounds as well as clear descriptions of the quantities of interest and utilisation of the expert-derived distributions [7, 8]. Examples such as this, identified within the review, will help improve the conduct and reporting of structured expert elicitation exercises in the future.

Several limitations of this review are noted, including the variable descriptions of elicitations in the broader literature and the lack of access to appraisal supplementary materials. Due to the varying description of structured expert elicitations within the broader literature, it may be that other studies in fact have used structured approaches but were not identified in the searches or screening due to a large omission of details. However, given the lack of survival outcome-specific guidance until now and the perceived challenge of conducting structured expert elicitations, we expect this impact to be minimal.

Whilst the identification of studies and NICE appraisals using structured expert elicitation is encouraging, the lack of specific methodological guidance for the elicitation of long-term survival outcomes appears to have led to variable implementation and adaptation of more general frameworks such as SHELF or the MRC protocol [13, 15]. With the recent publishing of the NICE TSD 26 for expert elicitation of long-term survival outcomes, we believe that the use of structured expert elicitation for survival outcomes will increase. This review will hopefully help to support this by acting as an early summary of optimal and less optimal implementations and designs of expert elicitation for long-term survival outcomes. We anticipate that guidance will continue to evolve as more structured elicitations are conducted. It will therefore be of great interest to continue to assess how this new guidance is received and implemented within health technology assessment more broadly.

## Conclusion

This review of the use of structured expert elicitation for long-term survival outcomes in the broader literature and NICE appraisals revealed that whilst experts are frequently consulted when extrapolating survival, structured elicitation approaches are not often used. In cases where structured methods are used, there appears to be considerable variability in conduct and reporting of the elicitation which leads to uncertainty in the validity and applicability of expert-derived estimates.

There is considerable scope for improvement in the methods and reporting of structured expert elicitation for long-term survival outcomes. This review serves as an initial summary of the current implementation and reporting of structured expert elicitation for long-term survival outcomes and highlights key areas for development and refinement.

## Supplementary Information

Below is the link to the electronic supplementary material.

Supplementary Material 1

## Data Availability

All data can also be accessed directly from the relevant publications [7, 8, 21, 23, 26, 27]. Direct access to the NICE technology appraisals is via https://www.nice.org.uk/guidance/published?sp=on.

## Abbreviations

NICE: National Institute for Health and Care Excellence
TSD: Technical Support Document
MRC: Medical Research Council
SHELF: Sheffield Elicitation Framework
QoI: Quantity of Interest
IDEA: Investigate, Discuss, Estimate, Aggregate
STEER: Structured Expert Elicitation Resources
PRISMA: Preferred Reporting Items for Systematic Reviews and Meta-Analyses
TA: Technology appraisal
