# Supplementary material for: Structured expert elicitation for long-term survival outcomes in health technology assessment: a systematic review
Source: BMC Med Inform Decis Mak. 2025 Oct 21;25:387. doi: 10.1186/s12911-025-03221-2 (PMC12539001; doi:10.1186/s12911-025-03221-2)
Supplement: Supplementary file 1 — Supplementary Material 1 [file 12911_2025_3221_MOESM1_ESM.docx]

# Supplementary material

## Search strategy for the broader literature

A search algorithm was designed for the database search using broad keywords for "expert elicitation" and synonyms combined with keywords for "survival". The term “expert” is a broader and umbrella term compared to “structured” and thus “structured” was not included explicitly within the search algorithm. It was expected that the keyword “expert” would co-occur with any reference to “structured”. Supplementary searches were carried out to identify studies not retrieved in the electronic database searches i.e. citation searching, reference tracking and hand-searching. The algorithm was adapted for the three databases including a limit to English only publications.

| **Ovid MEDLINE(R) Epub Ahead of Print and In-Process, In-Data-Review & Other Non-Indexed Citations and Daily July 24, 2024** | | **Results** |
| --- | --- | --- |
| # | Search term | 57837 |
| 1 | ((expert* or consensus) adj3 (opinion or elicit* or panel or judge* or estimat*)).tw. | 62842 |
| 2 | ((long-term or unobserved) adj3 (survival or model*)).tw. | 14670 |
| 3 | ((extrapolat* or probabilit*) adj3 survival).tw. | 76707 |
| 4 | 2 or 3 | 182 |
| 5 | 1 and 4 | 179 |
| 6 | limit 5 to english language | 57837 |
|  | | |
| **Embase 1974 to 2024 Week 32, 16^th^ August 2024** | |  |
| # | Search term |  |
| #1 | ((expert* or consensus) adj3 (opinion or elicit* or panel or judge* or estimat*)).tw. | 72482 |
| #2 | ((long-term or unobserved) adj3 (survival or model*)).tw. | 94690 |
| #3 | ((extrapolat* or probabilit*) adj3 survival).tw. | 21351 |
| #4 | 2 or 3 | 114633 |
| #5 | 1 and 4 | 280 |
| #6 | limit 5 to english language | 276 |
|  | | |
| **Web of Science, 25^th^ July 2024** | |  |
| # | Search term |  |
| 1 | ((expert* or consensus) NEAR/3 (opinion or elicit* or panel or judge* or estimat*)) (Title) or ((expert* or consensus) NEAR/3 (opinion or elicit* or panel or judge* or estimat*)) (Abstract) | 68,474 |
| 2 | ((long-term or "long term" or unobserved) NEAR/3 (survival or model*)) (Title) or ((long-term or "long term" or unobserved) NEAR/3 (survival or model*)) (Abstract) | 76,942 |
| 3 | ((extrapolat* or probabilit*) NEAR/3 survival) (Title) or ((extrapolat* or probabilit*) NEAR/3 survival) (Abstract) | 21,342 |
| 4 | #2 OR #3 | 97,235 |
| 5 | #4 AND #1 | 232 |
| 6 | #4 AND #1 and English (Languages) | 229 |

## List of included studies/NICE technology appraisals

| **Author/Appraisal number** | **Title** | **Year** |
| --- | --- | --- |
| Ayers *et al.* | Structured expert elicitation to inform long-term survival extrapolations using alternative parametric distributions: a case study of CAR T therapy for relapsed/ refractory multiple myeloma | 2022 |
| Cope *et al.* | Integrating expert opinion with clinical trial data to extrapolate long-term survival: a case study of CAR-T therapy for children and young adults with relapsed or refractory acute lymphoblastic leukemia | 2019 |
| Federico Paly *et al.* | Heterogeneity in Survival with Immune Checkpoint Inhibitors and Its Implications for Survival Extrapolations: A Case Study in Advanced Melanoma | 2022 |
| Klijn *et al.* | What Did Time Tell Us? A Comparison and Retrospective Validation of Different Survival Extrapolation Methods for Immuno-Oncologic Therapy in Advanced or Metastatic Renal Cell Carcinoma | 2021 |
| Konidaris *et al.* | Assessing the Value of Cemiplimab for Adults With Advanced Cutaneous Squamous Cell Carcinoma: A Cost-Effectiveness Analysis | 2021 |
| Willigers *et al.* | The Role of Expert Opinion in Projecting Long-Term Survival Outcomes Beyond the Horizon of a Clinical Trial | 2023 |
| TA917 | Daratumumab with lenalidomide and dexamethasone for untreated multiple myeloma when a stem cell transplant is unsuitable | Oct-2023 |
| TA954 | Epcoritamab for treating relapsed or refractory diffuse large B-cell lymphoma after 2 or more systemic treatments | Mar-2024 |
| TA967 | Pembrolizumab for treating relapsed or refractory classical Hodgkin lymphoma in people 3 years and over | May-2024 |
| TA975 | Tisagenlecleucel for treating relapsed or refractory B-cell acute lymphoblastic leukaemia in people 25 years and under | May-2024 |
